# Evaluation of Antioxidant, Xanthine Oxidase-Inhibitory, and Antibacterial Activity of *Syzygium cumini* Linn. Seed Extracts

**DOI:** 10.3390/plants14030316

**Published:** 2025-01-22

**Authors:** Jitendra Pandey, Nitesh Jaishwal, Mamta Jayswal, Deep Chand Gupta, Bishnu Dhakal, David Budean, Gopal Lamichhane, Hari Prasad Devkota

**Affiliations:** 1 Department of Pharmacy, Crimson College of Technology, Pokhara University, Devinagar-11, Butwal 32900, Nepal; jaishwalnitesh87@gmail.com (N.J.); jayswalmamta60@gmail.com (M.J.); g.deepchand01@gmail.com (D.C.G.); bdhakal877@gmail.com (B.D.); 2 Department of Chemistry, University of Hawai’i at Manoa, 2545 McCarthy Mall, Honolulu, HI 96822, USA; dbudean@hawaii.edu; 3 Department of Nutritional Sciences, Oklahoma State University, Stillwater, OK 74078, USA; lamichhanegopal1@gmail.com; 4 Graduate School of Pharmaceutical Sciences, Kumamoto University, Oe-honmachi 5-1, Chuo-ku, Kumamoto 862-0973, Japan; devkotah@kumamoto-u.ac.jp; 5 Headquarters for Admissions and Education, Kumamoto University, Kurokami, 2-39-1, Chuo-ku, Kumamoto 860-8555, Japan

**Keywords:** *Syzygium cumini* (L.) Skeels, antigout, wild fruits, total phenol, total flavonoid, seed extract

## Abstract

*Syzygium cumini* (L.) Skeels, commonly known as the Jamun or Indian blackberry, is a tropical evergreen tree native to the Indian subcontinent, and it belongs to the Myrtaceae family. This research aimed to assess the antibacterial properties of the extracts derived from *S. cumini* seed kernels and evaluate their total flavonoid content, total phenol content, total carbohydrate content, antioxidant capacity, and inhibitory effects on xanthine oxidase. Cold maceration was chosen for its ability to preserve thermolabile compounds and efficiently extract bioactive constituents with minimal energy and equipment requirement, with hexane and methanol employed as extraction solvents. The methanolic seed kernel extract of *S. cumini* showed the highest flavonoid (127.78 μg quercetin equivalent/mg dried extract vs. 21.24 μg quercetin equivalent/mg in hexane dried extract) and polyphenol content (153.81 μg gallic acid equivalent/mg dried extract vs. 38.89 μg gallic acid equivalent/mg in hexane dried extract), along with significant carbohydrate content (475.61 μg glucose equivalent/mg dried extract vs. 5.57 μg GE/mg in hexane dried extract). It also demonstrated potent antioxidant activity (IC_50_: 9.23 μg/mL; ascorbic acid: 5.10 μg/mL) and xanthine oxidase inhibition (IC_50_: 14.88 μg/mL), comparable to the standard drug allopurinol (IC_50_: 6.54 μg/mL), suggesting its therapeutic potential. Moreover, the methanolic extract of seed kernels exhibited strong antibacterial activity, with inhibition zones of 19.00 mm against *S. epidermidis*, higher than the standard antibiotic (gentamicin: 18.33 mm) against *K. pneumonia* (ciprofloxacin: 33.66 mm). The lowest minimum inhibitory concentration (MIC) and minimum bactericidal concentration (MBC) values of 0.32 mg/mL and 0.52 mg/mL, respectively, were observed for the same extract against *S. epidermis*. In conclusion, this study demonstrated the remarkable antibacterial effects of *S. cumini* methanolic seed kernel extract against various pathogenic microorganisms as well as significant inhibitory effects on xanthine oxidase and antioxidant activity.

## 1. Introduction

Plants can biosynthesize a large number of secondary metabolites, which are not only useful for maintaining their secondary functions but also increase plants’ value as nutraceuticals or functional foods, serving as source of lead compounds for drug discovery [1,2,3]. For instance, polyphenols are a major exogenous antioxidant in the plant-based diet, fighting against oxidative stress and inflammation [4,5]. This potential benefit was also observed by our ancestors, as evidenced by well-developed herbal medicinal systems, a long time ago. They used plants in the management of every kind of aliment, including infection, inflammation, and joint pain [6,7,8,9]. Although modern medicine has taken over the major stake of medicinal agents, nowadays, antibiotics resistance and side effects associated with modern medicines are major challenges necessitating more research to find safe and effective medicinal agents [10]. This makes plants potential candidates as alternative medicines and sources of lead compounds [11,12].

**Figure 1:**
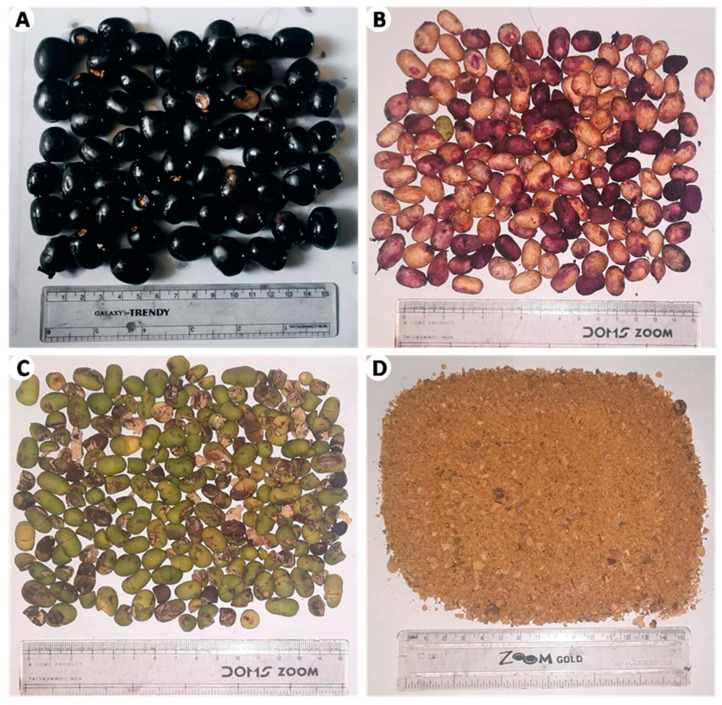
Photographs of *S. cumini*. (**A**) Mature and ripened fruits; (**B**) seeds from ripened fruits; (**C**) *S. cumini* seed kernels after removing seed coat; (**D**) dried powder of seed kernels.

*Syzygium cumini* (L.) Skeels, more commonly known as the Jamun or Indian blackberry, is a tropical evergreen tree native to the Indian subcontinent, and it belongs to the Myrtaceae family (Figure 1). This plant has several local names, such as Jambul, Jamun, Jambolao, Java plum, and black plum, and its fruit is a popular food in Southeast Asia [13]. It ripens from a green color to pink or crimson (Figure 1A). The ripe fruit can be eaten fresh, made into chutney or jam, or even processed in pickling to reduce its bitterness. This fruit is also used to make summertime drinks such as squash, sherbet, and syrup, in addition to being employed in the production of wine [14].

In addition to its culinary use, all parts of *S. cumini* has been utilized for centuries in various traditional medicine systems for their various healing properties [15]. Fruits of this plants are used to help with diabetes, pharyngitis, and splenic diseases. Seeds of the plant are used as diuretics and astringents and to manage diabetes [16]. Researchers have shown antidiabetic, hypoglycemic, antioxidant, anti-inflammatory, and antibacterial activities in the seed and fruit of this plant [17]. They have also reported the presence of bioactive compounds like jamboline, ellagitannins, gallic acid, and ellagic acid in the seeds of *S. cumini*.

While previous studies have investigated various aspects of *S. cumini* seeds, research remains limited on the detailed evaluation of MBC values against any bacterial strains and the MIC for specific pathogens, such as *B. cereus*, *S. pneumoniae*, and *S. enteritidis*. Additionally, the xanthine oxidase-inhibitory activity and proper quantification of total carbohydrate content of *S. cumini* seeds have not been reported yet. This study provides a comprehensive evaluation of *S. cumini* seed extracts, assessing their antioxidant, xanthine oxidase inhibitory, and antibacterial activities. By employing both hexane and methanolic extracts, we examined a wide range of pathogenic bacteria, including four Gram-positive and four Gram-negative strains. This integrated approach offers new insights into *S. cumini*’s diverse bioactivity, underscoring its potential for managing oxidative stress, gout, and bacterial infections and reinforcing its value for drug discovery.

## 2. Results

### 2.1. Extractive Yield Value

In this study, the extractive yields of *S. cumini* seed extracts using hexane and methanol were 4.62% and 8.36%, respectively, indicating efficient extraction. The 72 h triple cold maceration employed in this study resulted in a higher methanolic yield (8.36%) compared to a previous report of 5.9% from 24 h extraction [18] under similar conditions, demonstrating the benefit of prolonged extraction. Consistent with trends in other plants, such as licorice, where cold maceration (8%) outperformed Soxhlet extraction (6.5%) [11], these findings highlight the effectiveness of triple cold maceration in maximizing the recovery of bioactive compounds from *S. cumini* seeds.

### 2.2. Quantitative Measurement of Total Flavonoid and Total Phenolic Content

As shown in Table 1, the total flavonoid and phenolic contents in *S. cumini* seed extracts varied considerably based on the solvent used. For the hexane extract, the flavonoid content was 21.24 ± 1.73 µg QE/mg dry extract weight, and for the methanolic extract, it was substantially higher at 127.78 ± 0.89 µg QE/mg dry extract weight. Similarly, the total phenolic content in hexane and methanolic extracts of *S. cumini* seed kernel was 38.889 ± 1.05 µg GAE/mg and 153.81 ± 2.49 µg GAE/mg, respectively, emphasizing that polar solvents like methanol are far more efficient than non-polar solvents like hexane in extracting polyphenols. This significant difference (*p* < 0.05) highlights the importance of solvent polarity in maximizing the extraction of bioactive compounds, a finding that aligns with prior research indicating that polar solvents are generally more suitable for extracting polyphenols due to their compatibility with hydroxyl-rich molecules [19,20].

### 2.3. Quantitative Measurement of Total Carbohydrate Content

The results for total carbohydrate content are depicted in Table 1, where numerical data are presented as μg GE/mg of dried extract. As shown in Table 1, the total carbohydrate contents of *S. cumini* seed hexane and methanolic extract were 5.57 ± 0.57 and 475.61 ± 2.14 μg GE/mg dry extract weight, respectively.

### 2.4. Quantitative Measurement of Antioxidant Activity by Utilizing the DPPH Free Radical Inhibition Method

**Figure 2:**
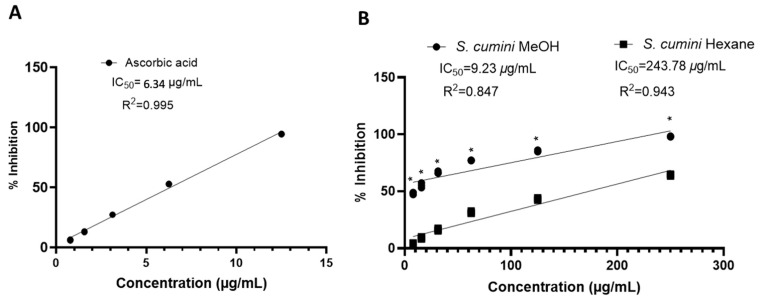
Dose–response scatter plot for ascorbic acid (**A**) and methanolic and hexane extract (**B**) under DPPH free radical scavenging activity assay. R^2^ represents linear regression coefficient of dose–response curve. Two-way ANOVA followed by Šídák’s multiple comparisons test was used to determine difference in free radical inhibition between methanol and hexane extract of *S. cumini*. * *p* < 0.0001 vs. hexane extract. The results are expressed as percentage of DPPH free radical scavenged by plant extract at the concentration of μg/mL.

As shown in Figure 2 and Table S1, the methanolic extract of *S. cumini* seed kernel demonstrated significant free radical inhibition, achieving a maximum of 98.07% inhibition at a concentration of 250 μg/mL. In contrast, the hexane extract exhibited only 64.34% inhibition at the same concentration. These results highlight the superior antioxidant activity of the methanolic extract, which is further reflected in its IC_50_ value of 9.23 μg/mL. This value is close to that of the standard ascorbic acid solution (IC_50_: 6.34 μg/mL), underscoring the methanolic extract’s high efficacy in scavenging free radicals. In comparison, the IC_50_ of the hexane extract was much higher at 243.78 μg/mL, suggesting a significantly lower antioxidant capacity.

### 2.5. Evaluation of Xanthine Oxidase Inhibitory Effect of S. cumini Seed Kernel Extracts

**Figure 3:**
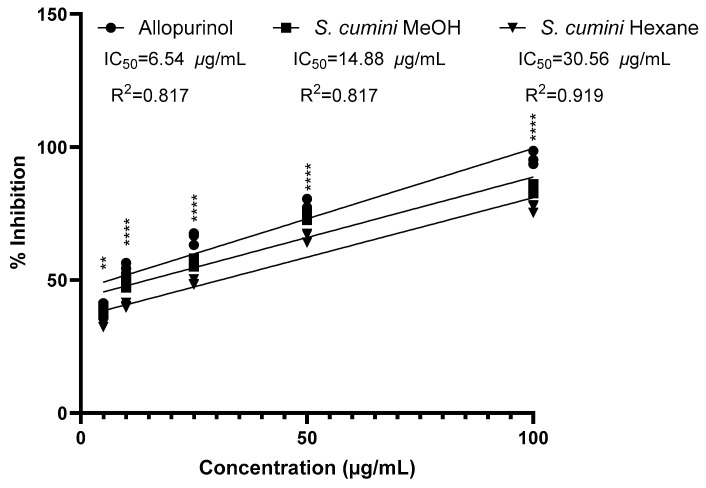
Dose–response scatter plot for allopurinol, methanol extract, and hexane extract under xanthine oxidase-inhibitory activity assay. R^2^ represents linear correlation coefficient of dose–response curve for respective treatment. Two-way ANOVA followed by Tukey’s multiple comparisons test was used to determine difference in free radical inhibition between allopurinol, methanol extract, and hexane extract of *S. cumini*. ** *p* < 0.01, and **** *p* < 0.0001 for methanol extract vs. hexane extract.

*S. cumini* seed kernels were assayed for xanthine oxidase-inhibitory effect and compared with the standard drug allopurinol at four different concentrations (Figure 3 and Table S2). The concentration of the extract at which it showed 50% of the xanthine oxidase-inhibitory effect (IC_50_) was also determined. All the analyzed samples exhibited an inhibitory effect in a dose-dependent manner. In general, the methanolic extract of the *S. cumini* seed kernel appeared to be more potent than the hexane extract, as it inhibited xanthine oxidase enzyme to a higher extent (84.32%) in comparison to hexane extract (77.05%) at the concentration of 100 μg/mL, which is comparable to the standard drug allopurinol (95.76%). All the results for the inhibition of xanthine oxidase shown by two different *S. cumini* seed extracts and standard allopurinol solution at different concentrations are presented in Table S2. Also, the IC_50_ value of the *S. cumini* methanolic extract was shown to be 14.88 μg/mL, which is comparable to the standard allopurinol (IC_50_: 6.54 μg/mL). However, the IC_50_ value of *S. cumini* hexane seed extract (30.56 μg/mL) was comparatively high.

### 2.6. Screening of Antibacterial Potency of S. cumini Seed Kernel Extracts

**Figure 4:**
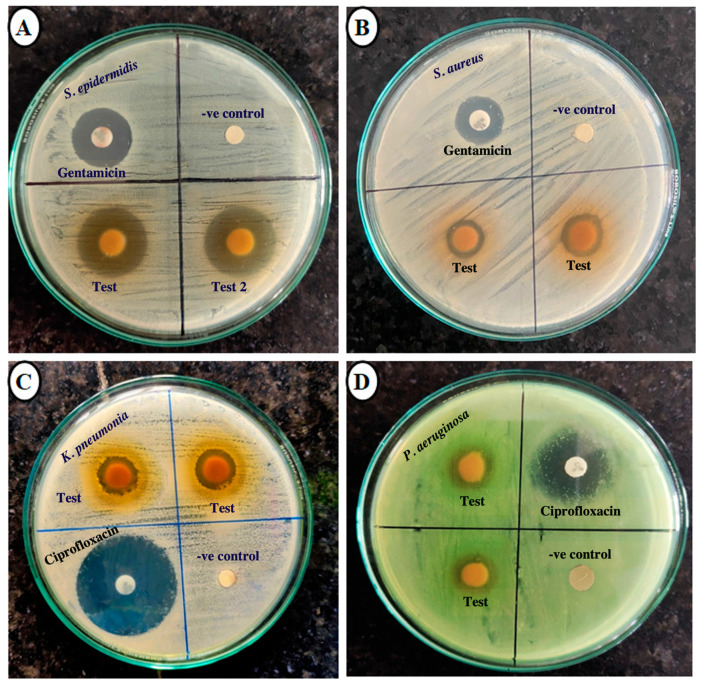
ZOI produced by *S. cumini* methanolic seed kernel extract against different pathogenic bacteria. (**A**) Against *S. epidermidis*; (**B**) Against *S. aureus*; (**C**) Against *K. pneumonia*; (**D**) Against *P. aeruginosa.* Note: 10% DMSO was used as negative control.

As shown in Table 2 and Figure 4, the methanolic extract of *S. cumini* seed kernel was most significant against *S. epidermis* (ZOI: 19.00 mm) and was even better than the standard drug gentamicin (ZOI: 18.66 mm). Moreover, this extract was moderately effective against all the Gram-negative bacteria as well. Among four different Gram-negative bacteria, it was most effective against *K. pneumoniae* (ZOI: 18.33 mm) but less active against *E. coli* (ZOI: 16.00 mm). However, its efficacy against *K. pneumoniae* was notably lower than the positive control ciprofloxacin (ZOI: 33.66 mm). In this way, methanolic extract of *S. cumini* seed kernel exhibited a moderate broad-spectrum antibacterial effect. In comparison to methanolic extract, the hexane extract of *S. cumini* seed kernel was less effective, and the lowest inhibitory effect was shown by this extract against *E. coli* (ZOI: 9 mm).

Both hexane and methanolic extracts of *S. cumini* seed kernel against eight different bacterial strains were further screened to evaluate their MIC and MBC capacity in terms of mg/mL. The MIC and MBC values of the screened extracts ranged from 0.32 mg/mL to 6.08 mg/mL and 0.5 mg/mL to 10.00 mg/mL, respectively. Among them, the methanolic extract exhibited the most potent activity against *S. epidermis*, with an MIC of 0.32 mg/mL and MBC of 0.52 mg/mL. Table 3 demonstrates that this extract was prominently effective against other examined Gram-positive bacteria as well as all the examined Gram-negative bacteria. The MIC (0.52 mg/mL) and MBC (0.78 mg/mL) values of this extract against Gram-negative strain *K. pneumonia* were almost comparable with its potency against Gram-positive strain *S. epidermidis*. Conversely, the hexane extract displayed the highest MIC and MBC values of 6.08 mg/mL and 10.00 mg/mL, respectively, against *E. coli*. Comparatively, both hexane and methanolic extracts depicted slightly better bactericidal effects against Gram-positive strains.

## 3. Discussion

### 3.1. Extractive Yield Value

The extraction of *S. cumini* seeds was carried out using methanol and hexane to ensure a comprehensive extraction of both polar and non-polar bioactive compounds, as these seeds are known to contain a diverse range of phytochemicals [15]. Methanol, a polar solvent, was selected due to its ability to dissolve phenolics and flavonoids by disrupting hydrogen bonding and enhancing solubility, resulting in a higher extraction yield and capturing compounds with known antibacterial properties [19,20,21]. Hexane, a non-polar solvent, was used to extract lipophilic bioactive compounds such as fatty acids, lipophilic vitamins (e.g., retinol, 3 IU/100 g), and phytosterols [22], and their antibacterial activity was documented in previous report [23]. In this way, the dual-solvent approach allowed the extraction of a wide spectrum of bioactive compounds, showing the contribution of those extracts to the antibacterial activity. Methanol demonstrated higher extraction efficiency, phytoconstituents quantity, and examined in vitro biological activities, while hexane provided insights into the role of non-polar compounds. This strategy ensured that both polar and non-polar compounds were included, enabling a thorough assessment of their relevance to antibacterial activity.

### 3.2. Quantitative Measurement of Total Flavonoid and Total Phenolic Content

Polyphenolic compounds, including flavonoids, are well known for their potent antioxidant properties. Flavonoids, a diverse subclass of polyphenols, exhibit antioxidant activity by neutralizing free radicals through hydrogen or electron transfer, largely due to the presence of hydroxyl groups in their structure [24,25]. This antioxidant mechanism allows flavonoids to effectively scavenge reactive oxygen species, singlet oxygen, and various free radicals, which helps in preventing diseases associated with oxidative stress, such as cancer, inflammation, cardiovascular diseases, neurological disorders, and immune dysfunction [26,27,28]. Similarly, polyphenols play a crucial role in medicinal plants due to their hydroxyl-rich structures, enabling effective scavenging of free radicals and providing significant therapeutic potential [29,30,31].

Compared to other studies, our results revealed a higher total flavonoid and phenolic content in *S. cumini* seed extracts. Previous studies have reported lower flavonoid content in *S. cumini* aqueous seed extracts, with values such as 10.11 mg catechin equivalent (CE)/g of dried seed powder [32] and 44.1 mg quercetin equivalent (QE)/g of dried seed extract [33]. In a related study, *S. cumini* seeds were extracted using the three-phase partitioning (TPP) technique, which employs ammonium sulfate, water, and butanol, whereas the upper butanol and lower aqueous phase were collected to obtain extract. In this study, the total flavonoid content of obtained extract was 7.78 CE/g [34]. However, our methanol-based extraction yielded a significantly higher flavonoid content, suggesting that methanol may be more effective than aqueous extraction methods. A similar study reported 233.8 mg QE/g in a hydro-methanolic extract [25], which is higher than our findings. This variation could be attributed to differences in solvent composition and extraction methods [25]. In terms of phenolic content, in a similar study, *S. cumini* seeds were extracted using TPP technique, and the total phenolic content obtained was 82.66 GAE/g [34]. Similarly, another comparable study reported 100.07 mg GAE/g in an aqueous *S. cumini* seed extract; both are lower than our methanolic extract findings, further supporting the effectiveness of methanol in polyphenol extraction [32]. Conversely, another study recorded a much higher phenolic content of 415 mg GAE/g [33] for an aqueous extract, possibly due to differences in extraction methods, geographical factors, or the specific part of the plant used [28].

Our study highlights the efficacy of methanol as an extraction solvent for maximizing yields of bioactive flavonoids and phenolic compounds from *S. cumini* seeds. These compounds include quercetin, rutin, epicatechin, kaempferol, myricetin, apigenin, and catechin [25,32,33] as well as gallic acid, tannic acid, ellagic acid, chlorogenic acid, caffeic acid, p-coumaric acid, rubuphenol, tetra-decamethyl-cycloheptasiloxane, and dodecamethyl-cyclohexasiloxane, and ferulic acid [35]. These compounds are known for their antioxidant, anti-inflammatory, and disease-preventive properties [32]. By optimizing extraction processes, our findings emphasize the therapeutic potential of *S. cumini* seed extracts for applications in nutraceutical and pharmaceutical formulations. The significance of using both methanol and hexane lies in their complementary polarity profiles, allowing for the comprehensive extraction of both polar and non-polar compounds [11,19,21]. This approach enables a broader understanding of the phytochemical diversity in *S. cumini* seeds and facilitates the identification of solvent-specific extraction efficiencies, contributing to optimizing the extraction process for targeted applications.

### 3.3. Quantitative Measurement of Total Carbohydrate Content

Carbohydrates are categorized under the large subclass of primary metabolites, which are bountifully synthesized in the plant cells during the photosynthetic pathway, and these metabolites always serve as a key element of every plant cell. For every metabolic pathway occurring inside living human cells, carbohydrates serve several functions, such as providing an indispensable source of energy, stimulation of insulin hormone release, serving as an essential chemical entity of neurotransmitters, and regulating the optimal serotonin concentration in living cells [36,37]. In recent days, several polysaccharides isolated from medicinal plants have shown diverse decisive biological activities [38].

From the data of total carbohydrate evaluation (Table 1), it is quite evident that extraction solvent has a major role in the extraction of carbohydrates. Methanol, a polar solvent, demonstrated significantly higher efficiency than the non-polar hexane (*p* < 0.05), highlighting substantial differences between the two extracts. These findings align with prior studies documenting the efficacy of polar solvents in isolating carbohydrates [27,36]. Interestingly, our findings differ from a previous study in India, which reported 89.68% carbohydrates in *S. cumini* seeds [39]. However, the methodology and sample state used in that study were not specified. Variations in carbohydrate content may be attributed to differences in extraction methods, sample preparation, or specific part of the seed analyzed [40]. For example, whole seeds versus seed kernel extracts may yield varying carbohydrate concentrations due to the presence of structural carbohydrates in seed coats or non-extractable bound forms [41]. Additionally, regional and environmental factors such as soil composition, climate, and agricultural practices can significantly influence the biochemical profile of *S. cumini* seeds [40].

Determining carbohydrate content is crucial for understanding the nutritional and therapeutic potential of *S. cumini* [39]. The carbohydrate-rich methanolic extract of *S. cumini* seed warrants further investigation to isolate and characterize bioactive polysaccharides and explore their pharmaceutical applications [42].

### 3.4. Quantitative Measurement of Antioxidant Activity by Utilizing the DPPH Free Radical Inhibition Method

The antioxidant activity of *S. cumini* seed kernel extracts is attributed to polyphenolic and flavonoid compounds, which neutralize free radicals via hydrogen donation and electron transfer [24]. In this study, when hexane and methanolic extracts were added to ethanolic DPPH solution, a decrease in absorbance was observed, which indicated DPPH radical reduction. The reduction in absorbance was then quantitatively measured to assess antioxidant efficacy [25]. The methanolic extract’s superior IC_50_ value highlights the role of polar polyphenol and flavonoid content, likely driving its enhanced antioxidant activity. Bioactive compounds in *S. cumini*—such as gallic acid, ellagic acid, catechin, epicatechin, ferulic acid, gallotannins, myricetin, quercetin, and kaempferol—are potent antioxidants that stabilize free radicals by donating electrons or hydrogen atoms, preventing cellular damage [25,43].

In a previous study conducted in India, the ethanolic seed extract of *S. cumini* demonstrated IC_50_ values of 8.6 μg/mL [43] and 14.0 μg/mL [44], while the hydromethanolic extract (70% methanol) exhibited an even lower IC_50_ of 5.1 μg/mL [28], indicating a higher antioxidant potential compared to our methanolic extract. Conversely, aqueous extracts reported IC_50_ values of 35.4 μg/mL [33] and 10.59 μg/mL [32], and the TPP extract presented an IC_50_ value of 12.15 μg/mL [34]. All those experiments were conducted by DPPH free radical scavenging method. These higher IC_50_ values indicate that the aqueous and TPP extracts possess lower antioxidant activity than our methanolic extract, further emphasizing the critical role of solvent choice in determining extraction efficiency and antioxidant capacity. Variations in IC_50_ values across studies may stem from differences in extraction methods and sample sources [15,32]

Despite the availability of studies on ethanolic, aqueous, hydromethanolic, and TPP solvents, very few studies have utilized methanol to extract *S. cumini* seeds. Therefore, we selected methanol as a polar solvent for its well-established efficiency in extracting phenolic and flavonoid compounds, and the results demonstrated considerable antioxidant activity. However, when compared to previous studies [28], our findings suggest that combining methanol with varying proportions of water may enhance the extraction of antioxidant constituents. This warrants further investigation to identify the optimal solvent system for maximizing the antioxidant potential of *S. cumini* seed extracts.

### 3.5. Evaluation of Xanthine Oxidase-Inhibitory Effect of S. cumini Seed Kernel Extracts

Xanthine oxidase (XO) is a widely distributed enzyme participating in the conversion of purine bases, and its excessive production is associated with gout development [45,46]. The treatment of gout primarily focuses on increasing the elimination of uric acid or reducing its production [47,48,49]. Drugs molecules such as allopurinol, uricosuric agents, corticosteroids, and NSAIDs are commonly used for gout management. Uricase enzymes offer potential advantages but face obstacles due to antibody susceptibility [50,51]. Xanthine oxidase (XO) inhibitors are increasingly attractive due to their reduced side effect profile compared to alternative agents. Allopurinol stands out as the primary XO inhibitor in clinical use despite its related adverse effects. Seeking other antigout treatments without undesirable effects, researchers have explored various plant species known for their traditional use in treating gout and arthritis [52,53].

Our investigation about the XO-inhibitory effect of *S. cumini* seed revealed the significant suppressive impact of the methanol-derived extract on xanthine oxidase inhibition. Extensive studies have highlighted the remarkable in vitro and in vivo xanthine oxidase-inhibitory effect of flavonoids and polyphenols such as quercetin [54], ferulic acid [55], kampferol [56], gallic acid [57], ellagic acid [58], epicatechin [59], caffeic acid [60], p-coumaric acid [61], and chlorogenic acid [62], which are abundantly present in *S. cumini* seeds [35]. Polyphenols and flavonoids inhibit xanthine oxidase (XO) through structural features that allow strong interactions with the enzyme’s active site. For polyphenols, the presence of multiple hydroxyl groups enables them to form hydrogen bonds with active-site amino acids, while their ability to chelate the molybdenum cofactor in XO enhances their inhibitory activity. Polyphenols with catechol or galloyl groups are particularly potent due to their capacity to disrupt the electron transfer in XO’s catalytic cycle. Similarly, flavonoids exhibit inhibitory effects due to a planar structure, such as a flavone backbone, and hydroxyl groups at specific positions, like C_3_, C_5_, and C_7_, which improve binding through hydrogen bonding and π–π interactions. These structural features collectively allow polyphenols and flavonoids to reduce XO activity, lowering uric acid production and oxidative stress [47].

This study is the first to report the xanthine oxidase-inhibitory activity of *S. cumini* seed kernel. In contrast, previous research has documented moderate XO-inhibitory activity in the methanolic extract of *S. cumini* leaves [63]. Differences in extraction conditions and plant parts can significantly influence phytochemical composition and, consequently, biological activity. Previous studies have demonstrated notable variability in the distribution and concentration of phenolics, flavonoids, and other secondary metabolites among different parts of *S. cumini* [15,35]. In comparison to the leaf, the seed kernel is rich in ferulic acid [35], ellagic acid [35] and gallotannins [64], which are bioactive compounds with strong XO-inhibitory activity [55,65]. Moreover, bioassay-guided extensive research should be conducted in different animal models to establish a scientific connection between the XO-inhibitory effect of *S. cumini* seed extract and its antigout effect.

### 3.6. Antibacterial Potency of S. cumini Seed Kernel Extracts

In this study, hexane and methanolic extracts of *S. cumini* seed kernel (1.5 mg dry extract/disc) were tested against eight pathogenic bacterial strains to evaluate their broad-spectrum antibacterial potential. The selected strains included Gram-positive bacteria (*S. aureus*, *S. pneumoniae*, *B. cereus,* and *S. epidermidis*) associated with respiratory, skin, and foodborne infections and Gram-negative bacteria (*E. coli*, *S. enteritidis*, *P. aeruginosa,* and *K. pneumoniae*) commonly linked to urinary, gastrointestinal, and hospital-acquired infections [66,67,68]. Antibacterial activity was quantified by measuring the inhibition zones using a Vernier caliper. As shown in Table 2, both extracts demonstrated greater efficacy against Gram-positive bacteria compared to Gram-negative strains. Usually, plant extracts exhibit more suppressive action against Gram-positive bacteria than Gram-negative strains due to availability of drug-impenetrable lipopolysaccharide architecture in the multi-layered cell wall in the Gram-negative bacteria, which is absent in Gram-positive strains. Additionally, Gram-positive bacteria possess a peptidoglycan layer that exhibits a mesh-like structure, making it more susceptible to the penetration of extracts [68,69].

The present study demonstrated that the methanolic extract of *S. cumini* seed kernel exhibited significant antibacterial activity, particularly against *S. epidermidis* (zone of inhibition, ZOI: 19 mm), exceeding the activity of the standard antibiotic gentamicin (ZOI: 18.66 mm), whereas the same extract was moderately active against all examined Gram-negative bacteria, with the highest ZOI (18.33 mm) against *K. pneumonia* (ciprofloxacin; 33.66 mm). These findings align with previous reports on the antibacterial efficacy of *S. cumini* seed extracts. For instance, an Indian study noted the moderate activity of ethanolic extracts against *S. aureus* (ZOI: 8 mm) and *E. coli* (ZOI: 9 mm), although the extract concentration was unspecified [70]. Similarly, methanolic extracts in another study exhibited greater inhibition against *Bacillus subtilis* (ZOI: 20.06 mm), albeit at a higher concentration (7.5 mg per well) [71]. Additionally, a single, unidentified compound isolated from the ethyl acetate fraction of *S. cumini* seed methanolic extract displayed ZOIs of 20 mm, 11 mm, 17 mm, and 15 mm against *E. coli*, *P. aeruginosa*, *S. aureus*, and *B. subtilis*, respectively, using the agar well diffusion method [72]. A study from Dhaka, Bangladesh, reported moderate antibacterial activity of the methanolic extract against *S. aureus* (ZOI: 10 mm) and *E. coli* (ZOI: 10 mm) [73]. Likewise, an aqueous extract containing 22.59 mg GAE/g exhibited a ZOI of 24.5 mm against *S. aureus* but showed no activity against *E. coli* and *P. aeruginosa* [74]. In another study in Nepal, ethanolic extracts demonstrated similar activity against Gram-negative *K. pneumoniae* (ZOI: 20 mm) and Gram-positive *S. aureus* (ZOI: 19 mm) [75]. An additional study from Tamil Nadu, India, using 800 µg of ethanolic extract per 6 mm well, reported ZOIs of 26, 20, 19, 17, and 17 mm against *P. aeruginosa*, *K. pneumoniae*, *Bacillus cereus*, *E. coli*, and *S. aureus*, respectively, which were significantly higher than the inhibition zones observed in our study [76].

The minimum inhibitory concentration (MIC) and minimum bactericidal concentration (MBC) values obtained in this study further underscore the broad-spectrum efficacy of methanolic extracts. The MIC value of 0.32 mg/mL against Gram-positive *S. epidermidis* and 0.52 mg/mL against Gram-negative *K. pneumoniae* highlights its potent antibacterial potential. These findings are consistent with previous studies from India and Brazil, which reported MIC values of 0.154 mg/mL to 0.648 mg/mL against *S. epidermidis* and *S. aureus*, respectively [77,78,79]. In contrast, an aqueous extract exhibited much higher MIC values of 1.41 mg/mL against *S. aureus* and 11.29 mg/mL against *E. coli* [74]. Interestingly, a study in Tamil Nadu, India, reported strikingly low MIC values for ethanolic seed extracts against *B. cereus* (<7.8 µg/mL), *P. aeruginosa* (15.6 µg/mL), *K. pneumoniae* (125 µg/mL), and *S. aureus* (125 µg/mL) [74]. These unusually low MIC values appear inconsistent with typical trends observed for plant extracts and warrant further investigation.

Overall, these discrepancies in antibacterial activity among different studies may be attributed to differences in the altitude of plant collection [80], experimental variation [81], extraction solvents [21], and extract concentrations used in prior studies, which were often unspecified. Our findings suggest that methanol might extract antibacterial compounds from *S. cumini* seeds more efficiently, as indicated by the comparative data. The prominent zones of inhibition exhibited by the unconfirmed isolated compound from the ethyl acetate fraction of the ethanolic extract in previous study [72] indicate that polar and semi-polar phytochemicals, which are efficiently extracted using ethanol and ethyl acetate [72], contribute to the significant inhibitory effect observed in the methanolic extract in this study. This hypothesis is consistent with the observation that methanol-based extractions consistently outperformed hexane in both ZOI and MIC/MBC analyses in our research. Moreover, numerous studies have demonstrated the potent antibacterial effects of flavonoids and polyphenols, both in vitro and in vivo. Key compounds exhibiting this activity include quercetin [82], ferulic acid [83], kaempferol [84], gallic acid [85], ellagic acid [86], epicatechin [87], caffeic acid [88], p-coumaric acid [89], and chlorogenic acid [90], all of which are abundantly found in *S. cumini* seeds [37]. The hexane extract, although less effective, exhibited measurable activity, particularly against *E. coli* (ZOI: 9 mm). This aligns with earlier findings that non-polar extracts, while generally less potent, may contain bioactive lipophilic compounds contributing to antimicrobial effects [70,72]. Importantly, the comparatively lower efficacy of hexane extract in our study highlights the importance of solvent selection in maximizing extract potency.

The novelty of our study lies in the comprehensive evaluation of MIC and MBC values across a wide range of pathogenic strains, including *B. cereus*, *S. pneumonia*, and *S. enteritidis*, which were not previously investigated. Notably, our study is the first to comprehensively evaluate the bactericidal activity of *S. cumini* hexane and methanol seed extract against both Gram-positive and Gram-negative pathogenic bacterial strains, advancing the understanding of *S. cumini* seed kernel’s bactericidal potential. Therefore, these findings not only expand the spectrum of antibacterial activity reported for *S. cumini* seed kernel extracts but also underscore their potential application as natural antibacterial agents. The methanolic extract’s consistent potency against both Gram-positive and Gram-negative bacteria suggests its potential for use in combating antibiotic-resistant pathogens. Further studies focusing on the isolation and characterization of active compounds are warranted to better understand the mechanisms underlying these effects and to develop standardized formulations for therapeutic applications.

In summary, significant differences in antibacterial, antioxidant, and xanthine oxidase-inhibitory activities between the methanolic and hexane extracts of *S. cumini* seeds can be attributed to the distinct phytochemical profiles of the extracts. The methanolic extract, rich in bioactive compounds such as polyphenols, flavonoids, and tannins, exhibits potent antimicrobial properties by disrupting bacterial cell walls and inhibiting enzymatic functions. In contrast, the hexane extract, with a lower yield, predominantly contains lipophilic compounds with limited bioactivity. These results underscore the effectiveness of polar solvents like methanol in extracting therapeutically relevant compounds, emphasizing the critical role of solvent selection in optimizing extraction processes for drug discovery research.

## 4. Materials and Methods

### 4.1. Chemicals Reagents, Solvents, and Standard Drugs

The reagents required in the study were Mueller Hinton Agar (MHA), gallic acid, nutrient broth, (HiMedia Laboratories Pvt. Ltd., Mumbai, India), 1,1-diphenyl-2-picrylhydrazyl (DPPH), aluminum chloride (AlCl_3_), sodium hydroxide (NaOH), sodium nitrite (NaNO_2_), and sodium carbonate (Na_2_CO_3_) (Thermo Fisher scientific, India Pvt. Ltd.; Mumbai, India); 3(4,5 dimethylthiazol-2-yl)-2-5-diphenyl tetrazolium bromide (MTT) (Beyotime Biotechnology, Haimen, China); quercetin, D-glucose, Folin–Ciocalteu reagent, dimethyl sulfoxide, phenol solution, H_2_SO_4_, and ascorbic acid (Thermo Fisher Scientific, India Pvt. Ltd., Mumbai, India); allopurinol (Himedia Laboratories Pvt. Ltd., Mumbai, India); uric acid (S.D fine–chem. limited, Mumbai, India); xanthine oxidase of bovine milk origin and xanthine (Sigma Aldrich, St. Louis, MO, USA); and barium chloride (HiMedia Laboratories Pvt. Ltd., Mumbai, India). Commercially available antibiotic discs of gentamycin and ciprofloxacin (Microxpress, A division of Tulip Diagnostics Pvt. Ltd., Goa, India) were utilized as positive control for antibacterial activity measurement.

### 4.2. Plant Sample Authentication

Ripened fruits of the *S. cumini* (Figure 1A) were collected from the Bhairahawa, Rupandehi, Nepal (tropical region, 544 m above sea level), in October 2022. The fresh fruits and herbarium of *S. cumini* were submitted to National Herbarium and Plant Laboratory Godabhari, Nepal (Ref no-079/080), for authentication. An authenticated herbarium of *S. cumini* was preserved in the pharmacognosy laboratory of the Crimson College of Technology, Nepal (Herbarium number: CCT/HRB/2022-03).

### 4.3. Extraction of S. cumini Seed Kernel

At first, only healthy and ripened fruits were washed using water. The pulp part of each fruit was removed manually with the help of a small knife to obtain the seed (Figure 1B). After manual removal of the seed coat, seed kernels were sliced into small pieces and spread over clean cotton cloth in the properly ventilated pharmacognosy lab for two weeks (shade drying). Dried samples of *S. cumini* seed kernels were subjected to comminution with the help of a grinder machine. Then, the comminuted fine powder was manually passed through a 40-mesh sieve. The seed kernel powder was then subjected to extraction in hexane (non-polar solvent) and methanol (polar solvent) separately. A simple and efficient triple cold maceration procedure was chosen to preserve the chemical integrity and bioactivity of thermolabile compounds by avoiding heat-induced degradation. This cost-effective, energy-efficient, and environmentally friendly method ensures compound stability, high recovery rates, and the retention of their natural profile [11]. For this, 100 g of *S. cumini* seed kernel powder was soaked with 1000 mL of hexane and methanol in a separate conical flask (total 200 g of powder for each solvent), with regular shaking every hour for 3 days. After 3 days, both samples were filtrated to collect the menstruum, and marcs were again soaked with the same volume of corresponding fresh solvents. This process was repeated in the same manner until the menstruum was collected three times. All the collected menstruum was well mixed and processed for solvent evaporation using a Rota evaporator (R-210/215, BUCHI Labor techno AG, Switzerland) at 40 °C to obtain the dried hexane and methanolic extract of *S. cumini* seed kernels. Dried extracts were stored at 4 °C until analysis.

### 4.4. Calculation of Extraction Yield

The extraction yield value of *S. cumini* seed in hexane and methanol was determined and expressed in percentage.

### 4.5. Measurement of Total Flavonoid Content

For the quantification of the total flavonoid content present in *S. cumini* seed kernel hexane and methanolic extract, a widely accepted aluminum chloride (AlCl_3_) method was adopted by following the protocols from the previous research article [12] with slight modifications. To plot the standard calibration curve, seven different concentrations of quercetin in ethanol (800 μg/mL to 25 μg/mL) were prepared by sequential serial dilution technique [18]. A plant extract sample solution of 1 mg/mL concentration was prepared for both hexane and methanolic extract by using ethanol (95% *v*/*v*) as diluents. After that, 2 mL of plant extract solution was properly mixed with 8 mL distilled water, followed by addition of 0.6 mL of 5% NaNO_2_, in a glass test tube. The mixture solution was agitated continuously for 5 min. After adding 0.6 mL of 10% AlCl_3_, the resultant sample mixture was subjected to incubation for 5 min. Furthermore, 4 mL of 1 molar NaOH solution was poured into the test tube, and the final mixture was again incubated for approximately 30 min at normal room temperature. The same process was repeated to prepare a blank sample, where 2 mL of ethanol (diluent) was used instead of the plant sample solution. Lastly, all the standard, plant samples, and blank solutions were analyzed in a UV–spectrophotometer (UV-1800 model; Shimazu Corporation Pvt Ltd., Shanghai, China) at 415 nm, in a triplicate manner [12]. The results are expressed as µg quercetin equivalent (QE) per mg of dry extract.

### 4.6. Measurement of Total Phenolic Content

For the quantification of total phenol content present in the *S. cumini* seed kernel hexane and methanolic extract, the widely accepted Folin–Ciocalteu (FC) method was adopted by following the protocols from the previous research article with slight modification. To plot the standard calibration curve, seven different concentrations of gallic acid in ethanol (800 μg/mL to 25 μg/mL) were prepared by sequential serial dilution technique [12]. A plant extract solution of 1 mg/mL concentration was prepared for both hexane and methanolic extract by using ethanol as the diluent. After that, 2 mL of plant extract solution was properly mixed with 2 mL FC reagent, followed by the addition of 10 mL water, in a glass test tube. The mixture solution shaken at 120 rpm continuously for 5 min. Furthermore, 2 mL of 10% Na_2_CO_3_ was poured into the test tube, and the final mixture was again incubated for approximately 1 h at normal room temperature. The same process was repeated to prepare a blank sample, where 2 mL of ethanol (diluent) was used instead of plant sample solution. Lastly, all the standard, plant samples, and blank solutions were analyzed in a UV–spectrophotometer at 725 nm, in a triplicate manner [12]. The results are expressed as µg gallic acid equivalent (GAE) per mg of dry extract.

### 4.7. Measurement of Total Carbohydrate Content

For the quantification of total carbohydrate content present in the *S. cumini* seed kernel hexane and methanolic extract, a widely accepted phenol sulfuric acid method was adopted by following the protocols from the previous research article with slight modifications. To plot the standard calibration curve, seven different concentrations of glucose in water (800 μg/mL to 25 μg/mL) were prepared by sequential serial dilution technique [36]. A plant extract sample solution of 1 mg/mL concentration was prepared for both hexane and methanolic extract by using water as the diluent. After that, 2 mL of plant extract solution was properly mixed with 1 mL of phenol solution (5% *v*/*v*) in a glass test tube. Furthermore, 5 mL of 10% H_2_SO_4_ was poured into the test tube gradually. The mixture solution was shaken at 120 rpm continuously for 10 min, and the final mixture was incubated in a water bath for 20 min at 30 °C. The same process was repeated to prepare a blank sample, where 2 mL of water (diluents) was used instead of the plant sample solution. Lastly, all the standard, plant samples, and blank solutions were analyzed in a U–spectrophotometer at 490 nm, in the triplicate manner [36]. The results are expressed as µg glucose equivalent (GE) per mg of dry extract.

### 4.8. Evaluation of Antioxidant Activity by Using DPPH Free Radical Inhibition Method

A widely accepted DPPH assay was chosen to evaluate the antioxidant potency of *S. cumini* seed kernel hexane and methanolic extract. All the procedure for this experiment was adopted from a previously published research article with a slight alteration [12]. DPPH solution having a concentration of 0.1 mM was prepared by using ethanol as diluents. Similarly, a stock solution of standard ascorbic solution (100 μg/mL) and plant extract solutions (1 mg/mL) were prepared by using the same diluents. After that, four different diluted concentrations of ascorbic acid solutions (12.500 μg/mL, 6.250 μg/mL, 3.1250 μg/mL, and 1.5620 μg/mL) and six different concentrations of plant extract solutions were prepared by serial dilution technique [12]. To carry out the free radical scavenging reaction, 5 mL of DPPH solution was transferred into a glass test tube followed by the addition of 5 mL plant extract solution into the same tube. This reaction was carried out for every diluted solution of plant extract and standard sample, in separate tubes. After mixing well, the resulting mixture solutions were kept in incubation for approximately 30 min, protecting them from the light.

Finally, the extent of DPPH free radicals scavenged by different concentrations of plant extract samples was monitored by measuring the absorbance of plant extract–DPPH solutions in a UV spectrophotometer at 517 nm. In this analysis, ethanol was used as blank control, and the ascorbic acid solution was used as a positive control. Every measurement was repeated three times. The results are presented as the percentage of DPPH free radicals inhibited by dried plant extract at different concentrations. Also, the IC_50_ (concentration of the sample in μg/mL that inhibit the 50% of DPPH free radicals) of ascorbic acid and plant extracts was determined through interpolation of the logarithmic regression analysis of the data obtained for different concentrations of plant extract [12].

### 4.9. Xanthine Oxidase-Inhibitory Activity

In vitro xanthine oxidase inhibitory assay was performed to investigate the in vitro antigout activity of the *S. cumini* seed kernel extracts by using the UV spectrophotometer method [91,92,93].

#### 4.9.1. Preparation of Xanthine, Xantine Oxidase (XO), and Sample Solution

At first, 0.15 mM of the xanthine solution was prepared by dissolving a small amount of NaOH, and the pH was maintained to 7.5. The xanthine oxidase solution (0.2 unit/mL) was adjusted to pH 7.5 by using 50 mM potassium phosphate buffer. Similarly, four different concentrations of test and standard samples (400, 200, 50 and 40 μg/mL) were prepared in 50 mM potassium phosphate buffer (pH 7.5) by serial dilution [91].

#### 4.9.2. Measurement of Xanthine Oxidase Inhibition

A total of 0.25 mL of standard and test solutions of four different concentrations (400, 200, 100, and 40 μg/mL) were mixed separately with 0.375 mL of xanthine solution and the same volume of 50 mM phosphate buffer (pH 7.5) to obtain the final concentration of 100, 50, 25, and 10 μg/mL. To initiate the reaction, 0.035 mL of XO solution was added and incubated at 25 °C for 10 min. The reaction was stopped by adding 0.1 mL of 1 M HCl. Finally, the change in absorbance was recorded at 295 nm by using a UV spectrometer. Allopurinol and phosphate buffer were used as standard and blank controls, respectively. The entire test was performed in triplicate. The results are presented as percentage of XO inhibited by dried plant extract at different concentrations (μg/mL) [93].

### 4.10. Antibacterial Activity Screening of S. cumini Seed Kernel Extracts

#### 4.10.1. Bacterial Strains

To scrutinize the in vitro antibacterial potency of hexane and methanolic extract of *S. cumini* seed kernel, four Gram-positive bacteria, namely *Staphylococcus aureus* (ATCC 25923), *Streptococcus pneumonia* (ATCC 49619), *Bacilus cereus* (ATCC-154872), and *S. epidermidis* (ATCC 12228), and four Gram-negative bacteria, namely *Escherichia coli* (ATCC 14948), *Salmonella enteritidis* (ATCC-155350), *Pseudomonas aeruginosa* (ATCC 15442), and *Klebsiella pneumoniae* (ATCC 4352), were purchased from Medicross Laboratory, Butwal, Nepal.

#### 4.10.2. Preparation of Plant Extracts and Filter Paper Discs

To prepare the *S. cumini* seed kernel hexane and methanolic extract solutions for antimicrobial analysis, 150 mg of each extract was transferred into a small vial and homogeneously solubilized with 1 mL of DMSO (10% in sterilized deionized water). Thus, prepared plant extract solution contained 1.5 mg of dry extract in each 10 μL of extract solution. By using a punching machine, filter paper discs of 5 mm diameter were punched out from Whatman’s No. 1 filter paper followed by sterilization in an autoclave at 115 °C for up to 15 min [12].

#### 4.10.3. Muller Hinton Agar (MHA) Media Preparation and Sub-Culturing of Bacterial Strains

The most common and scientifically accepted disc diffusion technique was chosen to screen the antimicrobial capacity of *S. cumini* seed kernel hexane and methanolic extract. MHA media was prepared by dissolving 19 g of MHA powder into 500 mL of distilled water. The homogenous solution of media prepared in a conical flask was closed tightly by using a clean cotton plug, and aluminum foil was wrapped over it. To sterilize the media solution, the conical flask was kept inside an autoclave for 15 min at the standard condition of temperature (121 °C) and pressure (15 lb). With the help of sterile laminar airflow, the sterile media was cooled up to 40–50 °C. After that, the media was aseptically transferred into several sterile Petri dishes and subjected to settlement. Randomly, two well-settled Petri dishes were selected for possible microbial contamination examination and incubated for 24 h by maintaining 37 °C. Other remaining Petri discs were preserved in a refrigerator at a temperature of 5 °C [94].

To subculture the ATCC strains of all the bacteria, a clean inoculating loop was heated over a Bunsen burner and dipped into different bacterial suspension tubes separately after cooling. Each inoculating loop, loaded with bacterial strain, was evenly splotched throughout the surface of the sterile MHA media Petri disc in a zigzag fashion. Overall, eight different bacteria were sub-cultured into different Petri discs and well labeled, and bacterial growth was allowed by incubating for a single day at 37 °C. For the whole experiment, the sterile and aseptic conditions of the entire environment were well maintained with laminar airflow [29].

#### 4.10.4. Preparation of Bacterial Suspension/Inoculums

Initially, nutrition broth media were made and sterilized, and 5 mL of broth media was aseptically transferred into eight different well labeled (each tube labeled with individual investigating bacterial strain) sterile test tubes. From the corresponding sub-cultured Petri discs, by using a sterile inoculating loop, all eight different bacterial strains were seeded into individual broth media test tubes. At last, all the sterile test tubes were transferred into an incubator for a single day, maintaining a constant temperature (37 °C), to grow the bacterial colonies of all eight bacteria. The turbidity of each bacterial suspension was compared with 0.5 McFarland solutions and adjusted to an adequate level [29,66].

#### 4.10.5. Exploration and Calculation of Zone of Inhibition (ZOI) Against Bacterial Strains

To depict the antibacterial potency of *S. cumini* seed kernel hexane and methanolic extract against eight different pathogenic bacteria strains, ZOI was calculated and is expressed in mm. For this, separate sterile cotton was dipped inside the different bacterial suspension tubes followed by streaking of each bacterial-loaded cotton stick over the whole surface of the distinct sterile Petri disc (MHA media loaded).

For this, a sterile cotton stick was dipped inside the labeled bacterial suspension tubes, followed by its smooth streaking over the whole surface of a sterile Petri dish (MHA media loaded). Several Petri dishes were loaded with the same bacterial strain, based on number of samples to be analyzed. Each bacterium-loaded Petri dish was labeled clearly with the name of the seeded bacteria. The same procedure was repeated for the other seven different bacteria. After completing the bacterial seeding for all the strains, each Petri dish was approximately separated into four equal regions. Ciprofloxacin (10 µg/disc) and gentamycin (10 µg/disc) were utilized as reference antibiotics for Gram-negative and Gram-positive bacteria, respectively. For testing the extracts, 10 µL of each extract, prepared at a concentration of 1.5 mg per disc, were applied to two separate paper discs in duplicate. A third disc, treated with 10 µL of DMSO, served as the negative control. All the analyses were performed three times. Finally, all the sample-loaded Petri dishes were incubated for a single day at 37 °C. On the next day, the area of bacterial growth, inhibited by investigating the plant extracts and standard antibiotic discs, was precisely measured with the help of a Vernier caliper [12,21,67]. The results are presented as ZOI in mm, shown by 1.5 mg of dried extract loaded on each filter disc.

#### 4.10.6. Calculation of MIC and MBC

To calculate the MBC and MIC of *S. cumini* hexane and methanolic seed extract against eight different pathogenic bacteria, a simple and widely accepted twofold serial broth microdilution method was chosen. For each extract, a total of 11 clearly labeled small-sized test tubes (nine for diluted extract solutions, one for blank control, and one for negative control) were sterilized. In each tube, 1.5 mL of sterile MHA broth media was transferred by using a micropipette. On other hand, to prepare nine different diluted concentrations of extract solution, nine different sterile test tubes were properly labeled. After that, 2 mL of stock solution (100 mg extract/mL) was prepared by using DMSO in the first test tube. The stock solution was then subjected to serial half dilution by using a diluent (DMSO and water mixture in equal ratio) to prepare the solutions of nine different dilutions (100 mg/mL- 0.390625 mg/mL). In the next step, 500 μL of plant extract solution was carefully poured into the corresponding broth media tube to yield nine different plant extract–broth media solutions having a concentration from 25 mg/mL to 0.09765 mg/mL (four-time dilution). Also, each vial was loaded with a bacterial inoculum of about 1 × 10^5^ CFU/mL. The turbidity of each bacterial inoculum was adjusted with that of 0.5 McFarland standards before transferring into test tubes. In the 10th tube, 500 μL of diluents was transferred and used as a blank control to check the sterility of the media. Similarly, only bacterial inoculum was transferred into the 11th tube and used as a negative control to confirm the suitability of broth media for bacterial growth. After that, all the samples were kept inside an incubator at 37 °C for 24 h. After 24 h, the MIC value of both extracts against six different bacteria was determined. The lowest concentration of plant extract (mg/mL) solution that was able to suppress the bacterial growth completely in the examined test tube was considered as the MIC. The survivability of the bacterial cells was monitored with the help of 3(4,5 dimethylthiazol-2-yl)-2-5-diphenyl tetrazolium bromide (MTT) by incubating for further 2 h at 37 °C, followed by visual examination of formazan formation.

For the calculation of MBC, which is the minimum concentration of plant extract (mg/mL) that can entirely kill the bacterial growth, preserved MHA Petri dishes (without any bacteria) were activated by incubating for 45 min at 37 °C and shifted into a sterile room supplied with continuous laminar airflow. After that, samples from every diluted test tube, which were prepared in the same way as in the MIC evaluation, were sub-cultured on activated MHA plates. Then, sub-cultured media were again transferred into an incubator for a single day by maintaining the constant temperature of 37 °C. At last, the lowest concentration of plant extract (mg/mL) sample that completely prevented bacterial growth over the media surface was finalized as the MBC [12,29,95,96].

### 4.11. Statistical Analysis

For every measurement, data were replicated three times and are expressed as mean ± SD. In the case of total phenol content, flavonoid content, and carbohydrate content determination, statistical significance of differences was calculated by one-way ANOVA and Tukey’s test. Similarly, for the analysis of antioxidant and xanthine oxidase inhibitory effect, Pearson correlation coefficient(r) was computed to find the linearity of the relationship between the dose of the extract and the DPPH-scavenged percentage. Statistical significance of the Pearson correlation was determined by calculating *p*-value using two-tailed *t*-test. Also, to observe the statistical significance of difference between hexane and methanol extract at the same dose, two tailed *t*-test was used. *p* < 0.05 was considered as statistically significant.

## 5. Limitations of the Study

While these results provide foundational insights, several limitations must be acknowledged. The lack of in vivo assessments precludes the evaluation of the extracts’ bioavailability, pharmacokinetic behavior, and safety parameters within a physiological framework. Furthermore, the reliance solely on bacterial cell lines limits the scope of understanding regarding the extracts’ potential effects in more intricate biological environments, such as those involving host immune mechanisms or other systemic interactions. The study was also constrained by the unavailability of advanced analytical methodologies, including high-performance liquid chromatography (HPLC) and gas chromatography (GC), which could have enabled a more precise characterization of the bioactive constituents. Additionally, while the antioxidant activity was evaluated using the 2,2-diphenyl-1-picrylhydrazyl (DPPH) assay, other complementary in vitro techniques, such as the 2,2′-azinobis-(3-ethylbenzothiazoline-6-sulfonic acid) (ABTS) radical scavenging assay, ferric reducing antioxidant power (FRAP) assay, and nitric oxide (NO) scavenging assay, were not performed. These additional methods could have provided a more comprehensive validation of the antioxidant effects of *S. cumini* seed extract. Furthermore, the absence of bioassay-driven fractionation hindered the identification of the specific molecules responsible for the observed biological properties. Future investigations should integrate multiple in vitro techniques, animal models, and clinical studies, alongside robust compound characterization and focused isolation strategies, to validate the pharmacological potential of *S. cumini* and elucidate its mechanisms of action in biologically relevant systems.

## 6. Conclusions

Our study highlights the promising therapeutic potential of *S. cumini* methanolic seed kernel extract as a multifunctional agent with notable antibacterial, antioxidant, and xanthine oxidase-inhibitory effects. The significant antibacterial activity observed against both Gram-positive and Gram-negative bacteria suggests that this extract could be a valuable alternative to combat multidrug-resistant pathogens associated with infections such as urinary tract infections, dental problems, dysentery, and diarrhea. This could be particularly relevant in addressing the global challenge of antimicrobial resistance by providing a natural, plant-based option. Furthermore, the xanthine oxidase inhibition exhibited by the extract points to its potential as an effective natural treatment for hyperuricemia and gout, as it may reduce uric acid synthesis. This finding could support the development of alternative therapies for gout management, particularly for individuals seeking natural remedies or those who experience side effects from conventional medications. The observed biological activities are likely attributed to the polyphenols and flavonoids present in the extract, which underscores the importance of *S. cumini* as a medicinal plant rich in bioactive compounds. However, we recommend further in vivo studies and molecular-level investigations to elucidate the mechanisms underlying its antibacterial and antigout effects. These studies will be essential in validating and expanding the potential applications of *S. cumini* seed kernel extract in the development of novel plant-derived therapeutic agents.

## Figures and Tables

**Table 1:** Results depicting the total flavonoid, phenol, and carbohydrate content from *S. cumini* hexane and methanolic seed kernel extract.

Extracts	Total Flavonoid Content (μg QE/mg Extract)	Total Phenol Content (μg GAE/mg Extract)	Total Carbohydrate Content (μg GE/mg Extract)
Hexane extract	21.24 ± 1.73 ^a^	38.89 ± 1.05 ^a^	5.57 ± 0.57 ^a^
Methanol extract	127.78 ± 0.89 ^b^	153.81 ± 2.49 ^b^	475.61 ± 2.14 ^b^

Note: All the data are represented as mean value ± standard deviation (n = 3). Different superscripts (a and b) within the column represent the significant differences (*p* < 0.05) in the seed content between hexane and methanol solvent.

**Table 2:** Zone of inhibition shown by *S. cumini* seed kernel hexane and methanolic extract against different pathogenic bacteria.

Zone of Inhibition in mm (Mean ± SD)				
Bacterial Strains	Hexane Extract	Methanol Extract	Ciprofloxacin	Gentamicin
*S. aureus*	9.56 ± 0.0.81	15.33 ± 0.57	-	16.63 ± 0.47
*S. epidermidis*	10.16 ± 1.04	19.00 ± 1.00	-	18.66 ± 0.57
*B. cereus*	9.33 ± 0.57	14.68± 0.51	-	16.85 ± 0.31
*S. pneumoniae*	10.00 ± 1.00	12.00 ± 1.00	-	15.00 ± 1.00
*E. coli*	9.00 ± 1.00	16.00 ± 1.00	23.16 ± 0.76	-
*K. pneumonia*	14.66 ± 1.10	18.33 ± 0.57	33.66 ± 1.52	-
*P. aeruginosa*	9.36 ± 0.70	16.33 ± 0.57	23.33 ± 1.52	-
*S. enteritidis*	9.53 ± 0.20	16.63 ± 0.55	22.56 ± 0.60	-

**Table 3:** MIC and MBC values shown by *S. cumini* seed kernel hexane and methanolic extract against different pathogenic bacteria.

MIC and MBC Values of Samples (mg/mL)				
Bacterial Strains	Hexane Extract		Methanol Extract	
	MIC	MBC	MIC	MBC
*S. aureus*	2.68 ± 0.97	4.66 ± 2.45	0.65 ± 0.22	1.04 ± 0.45
*S. epidermidis*	2.12 ± 0.97	2.69 ± 0.97	0.32 ± 0.11	0.52 ± 0.22
*B. cereus*	4.66 ± 2.45	7.5 ± 0.00	1.04 ± 0.45	1.56 ± 0.00
*S. pneumoniae*	2.12 ± 0.97	4.66 ± 2.45	2.60 ± 0.90	3.12 ± 0.00
*E. coli*	6.08 ± 2.45	10.00 ± 4.33	1.04 ± 0.45	1.30 ± 0.45
*K. pneumonia*	1.30 ± 0.45	2.12 ± 0.97	0.52 ± 0.22	0.78 ± 0.00
*P. aeruginosa*	4.66 ± 2.45	7.5 ± 0.00	2.08 ± 0.90	2.60 ± 0.90
*S. enteritidis*	4.66 ± 2.45	6.08 ± 2.45	1.30 ± 0.45	2.08 ± 0.90

## Data Availability

The data generated in this research are presented in the manuscript.

## Supplementary Materials

The following supporting information can be downloaded at https://www.mdpi.com/article/10.3390/plants14030316/s1. Table S1: DPPH free radical scavenging capacity of *S. cumini* seed kernel hexane and methanolic extract, along with standard ascorbic acid solution, at various concentrations. Table S2: Xanthine oxidase-inhibition capacity of *S. cumini* seed kernel hexane and methanolic extract, along with standard allopurinol solution, at various concentrations.
