# Supplementary material for: Evaluation of Antioxidant, Xanthine Oxidase-Inhibitory, and Antibacterial Activity of Syzygium cumini Linn. Seed Extracts
Source: Plants (Basel). 2025 Jan 22;14(3):316. doi: 10.3390/plants14030316 (PMC11820589; doi:10.3390/plants14030316)
Supplement: Supplementary file 1 [file plants-14-00316-s001.zip › plants-3364703-supplementary.pdf]

Supplementary Materials

Table S1. DPPH free radical scavenging capacity of *S. cumini* seed kernel hexane and methanolic extract along with standard ascorbic acid solution, at various concentrations.

	DPPH free radical inhibition by *S. cumini* seed kernel extracts							
Concentrations (μg/mL)	DPPH Scavenging activity (%)							
	7.812	15.625	31.25	62.5	125	250	r	P value
Hexane extract	4.34±0.43^{a}	9.07±0.72^{a}	16.31±0.78^{a}	31.43±0.89^{a}	43.17±0.73^{a}	64.34±0.44^{a}	0.97	0.0012
Methanol extract	48.19±0.62^{b}	55.24±0.72^{b}	66.65±0.16^{b}	77.13±0.36^{b}	85.43±0.78^{b}	98.07±0.39^{b}	0.92	0.0092
DPPH free radical inhibition by ascorbic acid solution (%)								
Concentrations (μg/mL)	0.781	1 .531		3.062	6.125	12.250	r	P value
Ascorbic acid	6.12± 0.45	12.89±0.31		27.43±0.17	52.67±0.27	94.35±0.23	0.99	0.0001

Note- Different superscripts (a and b) within the column represent the significant differences ($p<0.05$) in the DPPH radical scavenge of seed between hexane and methanol solvent. The r and p value on the table represent Pearson correlation coefficient (r) and Statistical significance of the Pearson correlation, respectively. These values depict the linearity of the relation between dose of the extract and DPPH scavenged percentage. The results are expressed as percentage of DPPH free radical scavenged by plant extract at the concentration of μg/mL.

Table S2. Xanthine oxidase inhibition capacity of *S. cumini* seed kernel hexane and methanolic extract, along with standard allupurinol solution, at various concentrations.

Xanthine oxidase inhibition by *S. cumini* seed kernel extracts and standard drug allopurinol							
Concentrations (μg/mL)		XO inhibitory activity (%)				r	p value
	5	10	25	50	100	0.095	0.012
Alopurinol	39.40±1.93	54.43± 0.46	65.82± 1.02	77.38±0.93	95.76±1.72		
Hexane extract	33.41±1.11	40.43±0.87^{a}	48.98±1.32^{a}	66.14±0.47^{a}	77.05±1.34^{a}	0.096	0.0092
Methanol extract	38.07±1.60	48.22±1.54^{b}	56.98±0.74^{b}	74.13±1.32^{b}	84.32±1.13^{b}	0.095	0.013

Note- Note- Different superscripts (a and b) within the column represent the significant differences ($p<0.05$) in the XO inhibition percentage of seed between hexane and methanol solvent. The r and p value on the table represent Pearson correlation coefficient (r) and Statistical significance of the Pearson correlation, respectively. These values depict the linearity of the relation between dose of the extract and XO percentage inhibition.
